# Beyond the ECG: Toward Patient-Centered Digital Triage for Acute Coronary Syndromes

**DOI:** 10.1016/j.jacadv.2026.103145

**Published:** 2026-08-12

**Authors:** Ioannis Skalidis, Lisa Simioni, Giulia S. Beretta, Stephane Cook, Mario Togni

**Affiliations:** Department of Cardiology, HFR – Fribourg Cantonal Hospital and University, Fribourg, Switzerland

Patient-related delays remain a major barrier to timely diagnosis and treatment in acute coronary syndrome (ACS), particularly when symptoms are ambiguous or initially underestimated. In this context, digital tools capable of combining clinical information with rapidly acquired electrocardiographic data may offer a complementary strategy for earlier risk recognition.

Shvilkin et al^1^ present an elegant proof-of-concept study evaluating a portable cable-free electrocardiogram (ECG) device integrated with clinical risk assessment for ACS prediction. The approach addresses an important and persistent challenge in ACS care: reducing patient-related delays while avoiding unnecessary emergency department use among low-risk individuals. By combining pre-existing atherosclerotic cardiovascular risk, symptom characteristics, and ST-vector loop parameters, the authors propose a diagnostic framework that more closely reflects Bayesian clinical reasoning than ECG interpretation alone.

Several aspects merit further discussion. First, the study enrolled patients after presentation to an emergency department or chest pain unit, with structured physician-assisted acquisition of clinical and ECG data. Although this design is appropriate for initial validation, the intended use case is patient-operated early self-triage before medical contact. Future studies should therefore clarify how model performance changes when symptom input and ECG acquisition are performed independently by patients, particularly among older individuals, patients experiencing anxiety during symptoms, or those with limited digital literacy.

Second, the improvement observed with individualized postevent reference ECGs is notable, with the pre-existing atherosclerotic cardiovascular risk and symptom risk model achieving the highest discrimination. However, the reference recording was obtained 9 to 12 months after the index event rather than before symptom onset. This raises the question of whether a true baseline ECG stored prospectively in a personal device would further improve specificity, especially in patients with baseline repolarization abnormalities, left ventricular hypertrophy, conduction disease, or prior myocardial infarction.

Third, the clinical implications of false-positive and false-negative trade-offs warrant careful prospective evaluation. At matched sensitivity, the reference-based model substantially reduced false positives compared with human clinical triage, suggesting potential value in limiting unnecessary urgent evaluations. However, in ACS triage, the acceptable threshold for missed non–ST elevation myocardial infraction (STEMI) or unstable angina may differ from that for STEMI. Future validation should therefore report performance separately across STEMI, non-STEMI, unstable angina, and noncardiac chest pain, ideally linked to downstream outcomes, time-to-treatment, and health care utilization.

Overall, this study provides an important early contribution to patient-centered digital ACS triage. External, multicenter, real-world validation will be essential to determine whether this approach can safely complement existing emergency care pathways.
